# A Formation Control Method for AUV Group Under Communication Delay

**DOI:** 10.3389/fbioe.2022.848641

**Published:** 2022-03-16

**Authors:** Yuepeng Chen, Xuan Guo, Guangyu Luo, Guangwu Liu

**Affiliations:** ^1^ School of Automation, Wuhan University of Technology, Wuhan, China; ^2^ Green Ship and Marine Engineering Equipment Technology, Wuhan University of Technology, Wuhan, China

**Keywords:** heterogeneous AUV group, consistency algorithm, leader–follower, communication delay, graph theory

## Abstract

This article presents a consistency control algorithm for the autonomous underwater vehicle (AUV) group combined with the leader–follower approach under communication delay. First, the six-degree-of-freedom (DoF) model of AUV is represented, and the graph theory is used to describe the communication topology of the AUV group. Especially, a hybrid communication topology is introduced to adapt to large formation control. Second, the distributed control law is constructed by combining the consensus theory with the leader–follower method. The consistency control algorithms for homogeneous and heterogeneous AUV groups based on the leader–follower approach under communication delay are proposed. Stability criteria are established to guarantee the consensus based on the Gershgorin disk theorem and Nyquist law, respectively. Finally, numerous simulation experiments are carried out to show the effectiveness and superiority of the proposed algorithms.

## 1 Introduction

AUV is a device that can perform various tasks underwater instead of a human (Shen et al., 2016; Huang et al., 2020; Lin et al., 2020; Sánchez et al., 2020; Zong et al., 2021). In general, complex underwater operations are usually accomplished by the AUV group. Compared to a single AUV, the AUV group owns powerful and comprehensive capabilities to accomplish complex tasks (Park et al., 2019; Connor et al., 2021; Hadi et al., 2021). Moreover, the formation control problem is of great interest in studying the AUV group. Various formation control approaches have been reported in the literature, such as the path-following approach (Xinjing et al., 2017; Yu et al., 2017), the leader–follower approach (Bechlioulis et al., 2019; Renjie et al., 2020; Wang et al., 2020; Heshmati-Alamdari et al., 2021; Zhang et al., 2021), the behavioral approach (He et al., 2010; Chen et al., 2021), the virtual structure approach (Zhang et al., 2016), and the consensus theory (Zhang et al., 2019; Qin et al., 2020; Xia et al., 2021b). The leader–follower method is widely used because of its simple structure and easy implementation, but it relies too much on the leader. If the leader fails, the entire formation system will collapse. Consensus theory usually assumes that AUVs only interact with their neighboring AUVs, which are suitable for large-scale formation control. However, it is difficult to find appropriate quantization information and topology to ensure that the consistency algorithm converges in a limited time. Meanwhile, there are time delays in the hydroacoustic communication among AUVs in an underwater environment. In this study, the consistency algorithm is combined with the leader–follower approach under communication delay to be applied in the formation control of the AUV group.

The formation control of the AUV group has received increasing attention from marine technology and control engineering communities. In Chen et al. (2020), a classification framework with three dimensions, including AUV performance, formation control, and communication capability, is proposed, which provides a comprehensive classification method for AUV formation research. In Xia et al. (2020), a distributed leader–follower control method is designed by combining the consensus theory and artificial potential field method (CMM-AUV) for the multi-AUV system with a leader. In this method, the communication delay is not taken into account. In Filaretov and Yukhimets (2020), a new path planning method for the AUV group in the leader–follower mode is proposed, which cannot be used in large formations because of a massive amount of information interaction. In Xia et al. (2021a), a dual closed-loop fast integral terminal sliding mode control method of the AUV group is proposed, which overcomes the problem that the formation tracking errors of the traditional method may not converge to zero in finite time. In this method, the communication topology is redundant and prone to message blocking. In Yuan et al. (2018), a new concept of formation learning control is introduced to the field of formation control of the AUV group without considering more realistic underwater control circumstances, including poor inter-AUV communication with time delays.

Although there are a large amount of studies on the AUV formation control field, critical issues still exist that have not been adequately addressed to date. In particular, formation control issues of the large AUV group need to be addressed. Specifically, realistic underwater control circumstances about communication delays need to be considered. Moreover, the heterogeneity of the large AUV group is not considered by most of the literature (Xia et al., 2021b). The contributions of this study are as follows:

1) A hybrid communication topology is established, which can be applied to the formation control of a large AUV group;
2) A distributed control by combining the consistency algorithm with the leader–follower method is achieved;
3) Consistent control of homogeneous and heterogeneous AUV groups while considering communication delay conditions is realized.

The rest of this study is organized as follows: Some preliminaries and modeling are introduced in Section 2. The consistency control method for the AUV group based on the leader–follower approach with communication delay is addressed in Section 3. Simulation results are provided in Section 4. The conclusions are drawn in Section 5.

## 2 Preliminaries and Modeling

### 2.1 Graph Theory

The graph theory is a powerful tool to deal with the consensus problem of multi-agent systems (Pratap et al., 2020; Zhang and Han, 2020). It is very effective to use graphs to represent the communication topology of information exchange among AUVs. Let us assume that every node in the graph corresponds to an AUV in our group, and the edges in the graph represent the information connection among AUVs. Therefore, the multi-AUV system can be referred to as a graph. The basic theory of graphs can be found in Ţurlea et al. (2019), which is omitted for simplicity.

A graph can be represented by an adjacency matrix *A*(*G*). This matrix is always square and has zero on its diagonal unless it is a loop. *A*(*G*) can be used to characterize the information interaction topology of the AUV group. The element’s value in *A*(*G*) is described as follows:

$$a_{kn}=\left\{\begin{array}{c} 1\left(v_{n},v_{k}\right)\in E\left(G\right)\;\\ 0,others\; \end{array}\right.,$$ (1)

where *G* is an undirected graph, and *A*(*G*) is a symmetric matrix with all zeros on the main diagonal. Generally, the weighted adjacency matrix *A*_*w*_(*G*) is defined as follows:

$$A_{w}\left(G\right)=\left[a_{kn}\right]=\left\{\begin{array}{c} w_{kn}\left(v_{n},v_{k}\right)\in E\left(G\right)\;\\ 0,others\; \end{array}\right..$$ (2)

The Laplacian matrix *L*(*G*) is another matrix that describes the relationship between nodes and edges in graph. The elements of *L*(*G*) are given by the following expression:

$$l_{kn}=-a_{kn},k\neq n,l_{kk}=\sum_{n=1}^{N}a_{kn},k=n.$$ (3)

The adjacency matrix and Laplacian matrix have the following remarkable properties:

Lemma 1. *Given a directed graph G and its adjacence matrix* *A*(*G*)*, if* *A*(*G*) *is irreducible, then* *G* *is a strongly connected graph.*

Lemma 2. *The rank of a strongly connected directed graph* *G* *with* *N* *nodes is* *rank*(*L*(*G*)) = *N* − 1*.*

Lemma 3. *A symmetric graph* *G* *is connected if and only if* *rank*(*L*(*G*)) = *N* − 1*.*

Lemma 4. *L*(*G*) *is positive semi-definite.*

Lemma 5. *If zero is the eigenvalue of* *L*(*G*)*, the graph is connected.* 1_*N*_ ∈ *R* ^*N*^ *is its corresponding eigenvector, where* 1_*N*_ = [1 … 1]^*T*^ *.*

Lemma 6. *The eigenvalues of the Laplacian matrix are always non-negative. Moreover, they can always be ordered as follows:*

$$0=\;\lambda_{1}\left(L\left(G\right)\right)<\lambda_{2}\left(L\left(G\right)\right)<\ldots ,<\lambda_{n}\left(L\left(G\right)\right).$$

### 2.2 Dynamic Model of AUV

**FIGURE 1:**
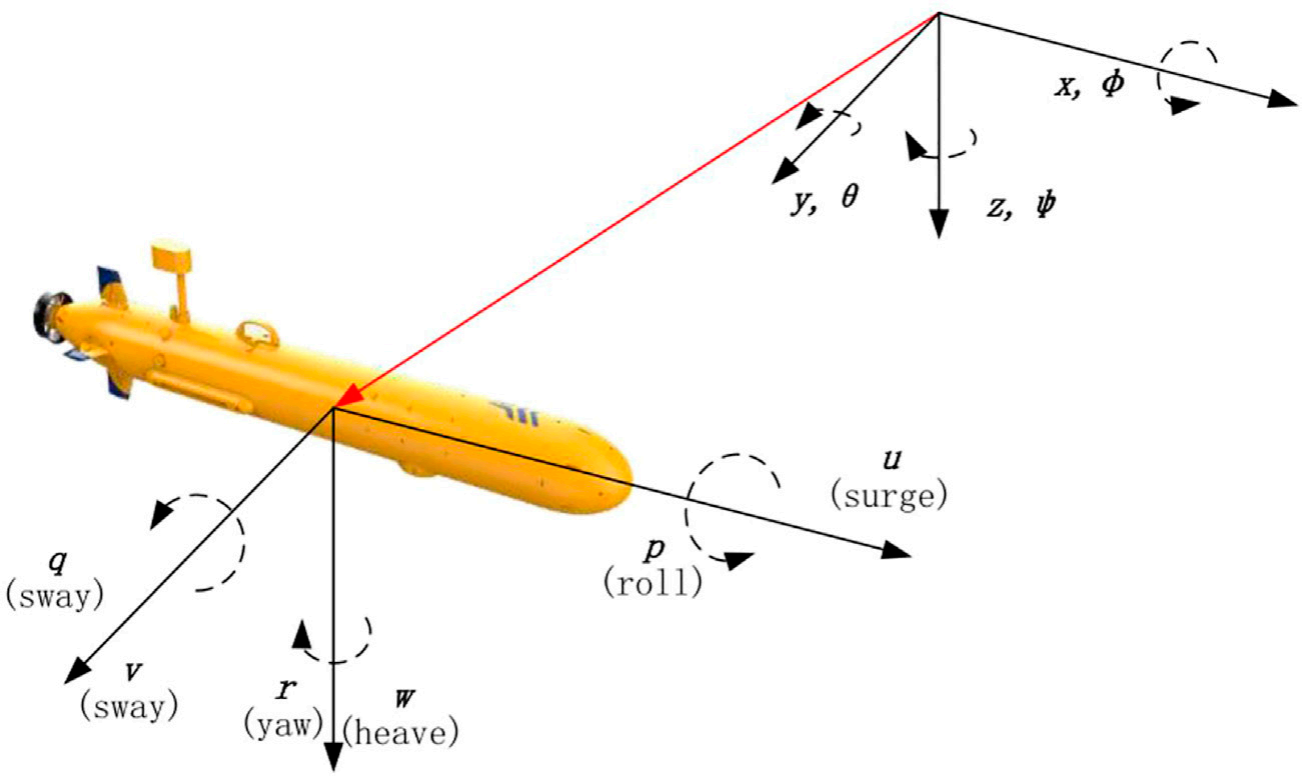
Model diagram of AUV in inertial and fixed coordinate systems.

Usually, the AUV dynamic model is divided into two parts: kinematics that only examines the geometric dimension of motion and kinetics that analyzes the forces that generate motion (Zhang et al., 2015; Cole et al., 2020; Franchi et al., 2020). Figure 1 presents an example of the AUV model, which includes dynamic variables in the body-fixed coordinate frame and its position relative to the inertial coordinate frame (Ma et al., 2020; Gao et al., 2021). Table 1 indicates different model variables defined as AUV’s motion behaviors in accordance with the Society of Naval Architects and Marine Engineers (SNAME) (Duan et al., 2020).

**TABLE 1:** Symbolic representation of the AUV model.

Degree of freedom	Position and Euler angle	Linear velocity and angular velocity	Force and moment
Surging	*X*	*u*	*X*
Swaying	*Y*	*v*	*Y*
Heaving	*Z*	*w*	*Z*
Rolling	*Φ*	*p*	*K*
Pitching	*Θ*	*q*	*M*
Yawing	*Ψ*	*r*	*N*

The relationship between velocity and acceleration is mainly considered by the AUV dynamic model. The shape of AUV is symmetrical from left to right and approximately symmetrical from top to bottom. The six-degree-of-freedom motion model of AUV can be expressed as follows (Sun et al., 2020, 2021):

$$\left\{\begin{array}{c} \dot{\eta }=\;J\left(\eta \right)\nu \;\\ M\dot{\nu }+C\left(\nu \right)\nu +D\left(\nu \right)\nu +g\left(\eta \right)=\tau \; \end{array}\right.,$$ (4)

where $\eta \in R^{6}$ is the spatial position and posture of AUV in the fixed coordinate system, $\nu \in R^{6}$ is the linear velocity and angular velocity of the AUV in the motion coordinate system, *J*(*η*) is a rotation transformation matrix from the motion coordinate system to the fixed coordinate system, *M* is the inertia matrix of the system (including additional mass), *C*(*ν*) is the Coriolis force matrix (including additional mass), *D*(*ν*) is the damping matrix, *g*(*η*) is the gravity/buoyancy and moment vector, and *τ* is the thrust and moment vector. The specific meanings of the vectors and matrices mentioned previously are as follows:

$$\begin{array}{cc} \eta & ={\left[\eta_{1},\eta_{2}\right]}^{T}\\ \eta_{1} & =\left[x,y,z\right]\\ \;\eta_{2} & =\left[\varphi ,\theta ,\psi \right], \end{array}$$ (5)

$$\begin{array}{cc} v & ={\left[v_{1},v_{2}\right]}^{T}\\ v_{1} & =\left[u,v,w\right],\;v_{2}=\left[p,q,r\right], \end{array}$$ (6)

$$\begin{array}{cc} \tau & ={\left[\tau_{1},\tau_{2}\right]}^{T}\\ \tau_{1} & =\left[X,Y,Z\right],\tau_{2}=\left[K,M,N\right], \end{array}$$ (7)

$$\left[\begin{array}{c} {\dot{\eta }}_{1}\\ {\dot{\eta }}_{2} \end{array}\right]=\left[\begin{array}{cc} J_{1}\left(\eta_{2}\right) & 0\\ 0 & J_{2}\left(\eta_{2}\right) \end{array}\right]\left[\begin{array}{c} v_{1}\\ v_{2} \end{array}\right],$$ (8)

$$\begin{array}{c} J_{1}=\left[\begin{array}{ccc} c\cos\psi \cos\theta & \cos\psi \sin\theta \sin\varphi -\sin\psi \cos\varphi & \cos\psi \sin\theta \cos\varphi +\sin\psi \cos\varphi \\ \sin\psi \cos\theta & \sin\psi \sin\theta \sin\varphi +\cos\psi \cos\varphi & \sin\psi \sin\theta \cos\varphi -\cos\psi \sin\varphi \\ -\sin\theta & \cos\theta \sin\varphi & \cos\theta \cos\varphi \end{array}\right]\\ J_{2}=\left[\begin{array}{ccc} c1 & \;\sin\varphi t\theta & \cos\varphi \tan\theta \\ 0 & \cos\varphi & -\sin\varphi \\ 0 & \frac{\sin\varphi }{\cos\theta } & \frac{\cos\varphi }{\cos\theta } \end{array}\right],\theta \neq \pm \frac{\pi }{2} \end{array}.$$ (9)

The six-degree-of-freedom motion equation of AUV can be expressed as follows:

$$\begin{array}{cc} X & =m\left[\dot{u}-vr+wq-x_{G}\left(q^{2}+r^{2}\right)+y_{G}\left(pq+\dot{r}\right)\right.\left.+z_{G}\left(pr+\dot{q}\right)\right],\\ Y & =m\left[\dot{v}-wp+ur+x_{G}\left(pq+\dot{r}\right)-y_{G}\left(r^{2}+p^{2}\right)\right.\left.+z_{G}\left(qr+\dot{p}\right)\right],\\ Z & =m\left[\dot{w}-uq+vp+x_{G}\left(rp+\dot{q}\right)+y_{G}\left(rq+\dot{p}\right)\right.\left.-z_{G}\left(p^{2}+q^{2}\right)\right],\\ K & =I_{xx}\dot{p}+\left(I_{zz}-I_{yy}\right)qr+m\left[y_{G}\left(\dot{w}-uq+vp\right)\right.\left.-z_{G}\left(\dot{v}-wp+ur\right)\right],\\ M & =I_{yy}\dot{q}+\left(I_{xx}-I_{zz}\right)rp+m\left[z_{G}\left(\dot{u}-vr+wq\right)\right.\left.-x_{G}\left(\dot{w}-uq+vp\right)\right],\\ N & =I_{zz}\dot{r}+\left(I_{yy}-I_{zz}\right)pq+m\left[x_{G}\left(\dot{v}-wp+ur\right)\right.\left.-y_{G}\left(\dot{u}-vr+wp\right)\right]. \end{array}$$ (10)

### 2.3 Communication Modeling

**FIGURE 2:**
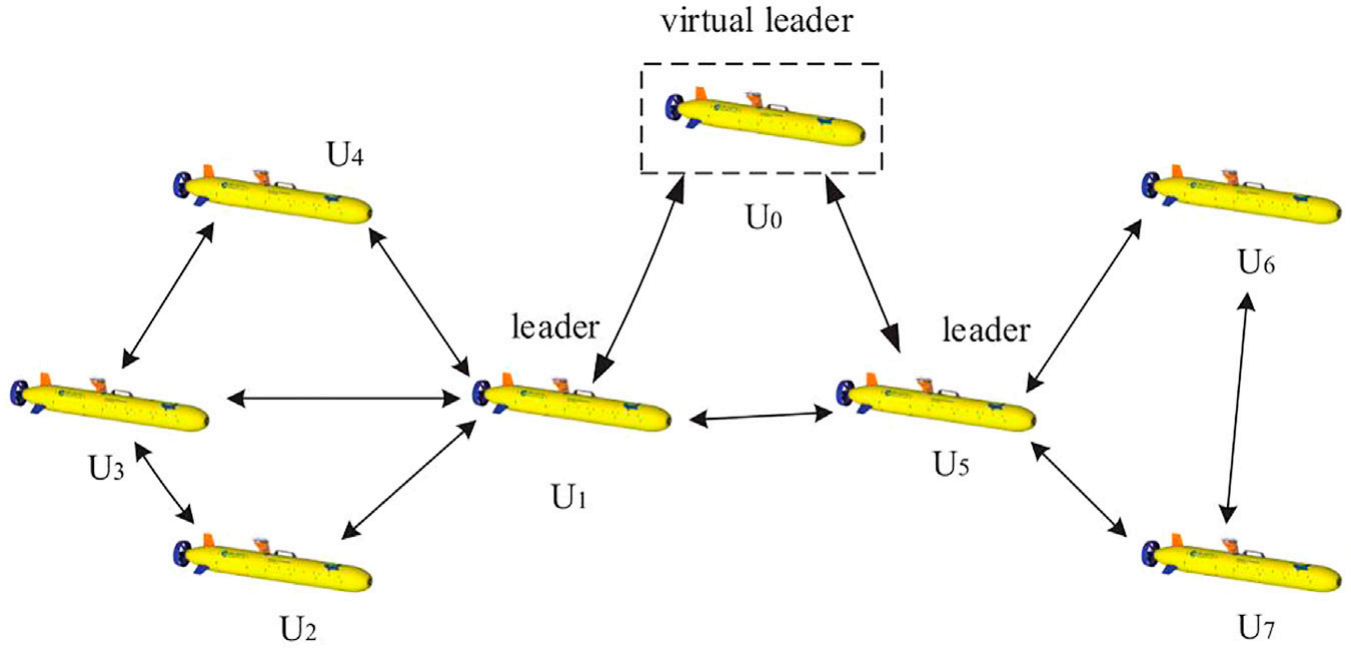
Hybrid communication topology.

At present, only one leader is defined in a group in most cases. The consistency process of the system is controlled by controlling the information interaction between the leader and other AUVs. In this study, multiple leaders and followers in the topological structure are adopted. The AUV group is divided into multiple small groups to form a swarm network. Each group that includes multiple AUVs has its own leader AUV. Moreover, there is one and only one AUV in the first group as virtual AUV, which is named virtual leader. The dynamic and kinematic characteristics of the virtual leader and the real AUV are the same. The desired speed and desired position of the follower are given as control inputs of the virtual leader. In other words, a mixed-mode topology jointly is designed to solve the consistency problem of the AUV swarm system. The schematic diagram of the topology is shown in Figure 2.

The network topology of AUV clusters is described by a directed graph *G* = (*V*, *E*). The set of vertices $V=\left\{v_{1},v_{2}\cdot \cdot \cdot ,v_{n}\right\}$ represents the AUV clusters. The set of edges *E* ⊂ *V* × *V* represents their interaction relationships. All AUVs in the system are divided into *m* + 1 clusters, which are denoted by *β*_0_, *β*_1_, … *β*_*m*_. There is a special AUV in each cluster called the pilot vessel, denoted by $l_{i}\in \beta_{i},\forall i\in \left\{0,1,\ldots ,m\right\}$ . The other AUVs in the cluster are denoted by *u*_*j*_. Among them, there is only one AUV in cluster *β*_0_, and the node is the virtual leader specifically denoted as $\beta_{i}=\left\{l_{i},u_{i,1},\ldots u_{i,n}\right\},\forall i\in \left\{1,\ldots ,m\right\}$ .

The set of pilot boats is denoted by $L=\left\{l_{0},l_{1},\ldots l_{m}\right\}$ . In the whole communication topology, only *l*_0_ has information transfer with the pilot boats of other clusters, and the pilot boats complete the information exchange between groups. $\left\{l_{1},\ldots ,l_{m}\right\}$ not only has inter-group information transfer and interaction with other pilot boats but each pilot boat also has intra-group information transfer and interaction with other nodes in the cluster to which it belongs. When the pilot boat communicates and interacts with the nodes in the cluster, the locally consistent equilibrium point of the cluster is periodically reset.

It is important to note that data transmission between AUVs is best done digitally so that there is no interference due to communication. Some data will be lost or miscoded during transmission, but no new interference will be introduced as in the case of analog transmission. In addition, each machine should add a GPS clock when sending data. When other AUVs receive the data, they unpack the data and decode the GPS clock when the data are sent, and compare it with the current GPS clock to know the size of the transmission delay. After avoiding the generation of transmission interference and accurately knowing the communication delay, the formation control accuracy and the control effect can be improved.

## 3 Controller Design

In this section, the motion controller of the homogeneous AUV group is designed based on the consensus theory and the leader–follower method so that all the followers can follow the leader for motion under communication delay. Furthermore, a distributed controller is designed for each heterogeneous AUV with a communication time delay.

### 3.1 Consensus Control Algorithm for Homogeneous AUV Group Under Communication Delay

In the actual engineering environment, the communication between AUVs underwater relies on hydroacoustic communication, the unreliability of communication channels, packet loss of communication data, failure of sensors and sonar equipment, the existence of communication barriers, and other factors, thus leading to intermittent communication interactions between AUVs, and there is often a certain time delay when AUVs receive position and velocity information. Therefore, considering that the information transmission between AUV individuals through sensors or communication devices will inevitably generate a time delay problem, the time delay in the process of information transmission from individual *i* to individual *j* is denoted by *τ*_*ij*_. If the time delay of the AUV’s own state is the same size as the communication time delay of the received information from its neighbors, a symmetric consistency algorithm is usually used as $u_{i}=-\underset{j\in N}{\sum }a_{ij}\left(t\right)\left(x_{i}\left(t-\tau_{ij}\right)-x_{j}\left(t-\tau_{ij}\right)\right)$ .

The control process of AUV swarm often needs to end in a finite time when it completes its assigned mission task, and the existence of communication time delay *τ*_*ij*_ affects the stability of the system and the convergence time of the system. Therefore, in order to ensure that the AUV swarm achieves asymptotic stability in finite time and realize the practical application of the system, it is necessary to design the upper limit of time delay *τ*_*ij*_ ≤ *τ*_0_ in order to study the consistency of the AUV swarm under the condition of time-varying communication time delay with fixed upper limit.

Furthermore, the dynamic of the tracking signal is described as follows:

In general, the motion of the AUV in water can be regarded as the spatial motion of a rigid body in a fluid. When the heading of the AUV in motion is constant and only the depth is changed, the center of gravity of the AUV is always kept in the same plumb plane within its changing heading and not changing depth. The motion model in 2.3.1 is adopted to ignore the motion of the cross-rolling surface, and the spatial motion in the water is approximated and decomposed into a horizontal plane motion and a vertical plane motion. Usually, the plane motion can reflect the basic characteristics of motion–depth and heading control, and the hydrodynamic characteristics of space motion are also developed on the basis of plane motion.

Suppose the AUV group is finally kept at the same depth, all the AUVs follow the leader AUV to keep a fixed formation, and only horizontal motion is considered. At this time, the position information of the first AUV is $\eta_{i}=\left(x_{i},y_{i},z_{i}\right)$ , and the velocity information is *v*_*i*_. Let the final horizontal surface speed of the AUV group be *v*_0_, the angular velocity of rotary motion be *r*_0_, and the vertical velocity be $v_{z_{0}}$ . Then, according to the AUV motion model established previously, the *i* AUV motion model can be simplified as follows:

$$\begin{array}{cc} {\dot{x}}_{i} & =v_{i}\cos\psi_{i}\\ {\dot{y}}_{i} & =v_{i}\sin\psi_{i}\\ {\dot{\psi }}_{i} & =r_{i}\\ {\dot{v}}_{i} & =\frac{1}{\lambda_{v_{i}}}\left(v_{0}-v_{i}\right)\\ {\dot{r}}_{i} & =\frac{1}{\lambda_{r_{i}}}\left(r_{0}-r_{i}\right)\\ {\dot{z}}_{i} & =v_{z_{i}}\\ {\dot{v}}_{z_{i}} & =\frac{1}{\lambda_{z_{i}}}\left(v_{z_{0}}-v_{z_{i}}\right), \end{array}$$ (11)

where *λ* is a parameter greater than zero. Let $\left(x_{f_{i}},y_{f_{i}}\right)$ denote the position of the point *f*_*i*_ on the *i* AUV at a distance *d*_*i*_ from the center of gravity $\left(x_{i},y_{i}\right)$ . Then, the simplified control algorithm using $\left(x_{fi},y_{fi}\right)$ instead of $\left(x_{i},y_{i}\right)$ in the coordinated control of the AUV is expressed as follows:

$$\left[\begin{array}{c} x_{f_{i}}\\ y_{f_{i}} \end{array}\right]=\left[\begin{array}{c} x_{i}\\ y_{i} \end{array}\right]+\left[\begin{array}{c} d_{i}\cos\psi_{i}\\ d_{i}\sin\psi_{i} \end{array}\right],$$ (12)

where $v_{0},r_{0},v_{z_{0}}$ satisfies

$$\left[\begin{array}{c} v_{0}\\ r_{0} \end{array}\right]=\left[\begin{array}{c} v_{i}\\ r_{i} \end{array}\right]+\left[\begin{array}{cc} \lambda_{v_{i}} & \\ & \lambda_{r_{i}} \end{array}\right]{\left[\begin{array}{cc} \cos\psi_{i} & -d_{i}\sin\psi_{i}\\ \sin\psi_{i} & d_{i}\cos\psi_{i} \end{array}\right]}^{-1}\left[\begin{array}{c} u_{x_{i}}+v_{i}r_{i}\sin\psi_{i}+d_{i}r_{i}^{2}\cos\psi_{i}\\ u_{y_{i}}-v_{i}r_{i}\cos\psi_{i}+d_{i}r_{i}^{2}\sin\psi_{i} \end{array}\right],$$ (13)

$$v_{z_{0}}=v_{z_{i}}+\lambda_{z_{i}}u_{z_{i}}.$$ (14)

We can get

$$\begin{array}{cc} {\dot{x}}_{f_{i}} & =v_{x_{i}}\\ {\dot{y}}_{f_{i}} & =v_{y_{i}}\\ {\dot{z}}_{i} & =v_{z_{i}}\\ {\dot{v}}_{x_{i}} & =u_{x_{i}}\\ {\dot{v}}_{y_{i}} & =u_{y_{i}}\\ {\dot{v}}_{z_{i}} & =u_{z_{i}} \end{array}.$$ (15)

Therefore, coordinated consistency control of the AUV cluster can be achieved by designing the control inputs $u_{x_{i}}$ , $u_{y_{i}}$ and $u_{z_{i}}$ , which means the tracking signal of the leader is $u_{x_{i}}$ , $u_{y_{i}}$ , and $u_{z_{i}}$ . It is assumed that each AUV has a unique number and that each AUV knows its own, as well as the numbers in its neighborhood. In addition, all interactions among AUVs are synchronized, that is, all update their state parameters at the same time.

Since there is a hydroacoustic communication delay among AUVs in the underwater environment, the consistency control algorithm for the AUV under the time delay condition is considered later. Similarly, assuming that the AUV group maintains a fixed depth motion when there is a time delay in the inter-AUV communication, the controller is designed as follows:

$$\begin{array}{cc} u_{x_{i}} & ={\dot{f}}^{x}\left(t\right)-\gamma (v_{x_{i}}\left(t-\tau_{ij}\left(t\right)\right)-f^{x}\left(t-\tau_{ij}\left(t\right)\right)\\ & -\overset{m}{\underset{j=1}{\sum }}a_{ij}\left[(x_{f_{i}}\left(t\right)-x_{f_{j}}\left(t-\tau_{ij}\left(t\right)\right)-\left(\delta_{i}^{x}-\delta_{j}^{x}\right)\right]\\ & -\overset{m}{\underset{j=1}{\sum }}a_{ij}\left[\gamma \left(v_{x_{i}}\left(t-\tau_{ij}\left(t\right)\right)-v_{x_{j}}\left(t-\tau_{ij}\left(t\right)\right)\right)\right], \end{array}$$ (16)

$$\begin{array}{cc} u_{y_{i}} & ={\dot{f}}^{y}\left(t\right)-\gamma (v_{y_{i}}\left(t-\tau_{ij}\left(t\right)\right)-f^{y}\left(t-\tau_{ij}\left(t\right)\right)\\ & -\overset{m}{\underset{j=1}{\sum }}a_{ij}\left[y_{f_{i}}\left(t\right)-y_{f_{j}}\left(t-\tau_{ij}\left(t\right)\right)-\left(\delta_{i}^{y}-\delta_{j}^{y}\right)\right]\\ & -\overset{m}{\underset{j=1}{\sum }}a_{ij}\left[\gamma (v_{y_{i}}\left(t-\tau_{ij}\left(t\right)\right)-v_{y_{j}}\left(t-\tau_{ij}\left(t\right)\right)\right]. \end{array}$$ (17)

where control gain *γ* > 0, *τ*_*ij*_(*t*) denotes communication time delay between AUV*i* and AUV*j*, *f* ^*x*^(*t*) and *f* ^*y*^(*t*) are continuous differentiable functions that denote the velocity characteristics of the AUV motion, and $\delta_{i}^{x}$ , $\delta_{i}^{y}$ denote the desired position. When the upper limit of time delay is *τ*_0_, 0 < *τ*_*ij*_(*t*) < *τ*_0_. Suppose that the communication between any two different AUVs allows a common upper limit of time delay *τ*_0_, that is, 0 < *τ*_*ij*_ ≤ *τ*_0_.

Theorem 1. *For AUV group with a directionless connected network topology, the control inputs are designed to be Eq.* 16 *and Eq.* 17*, respectively, and the communication time delay between its individuals* *τ*_*ij*_(*t*) *satisfies*

$$0\leq \tau_{ij}\left(t\right)\leq \frac{1}{\omega_{0}}\arctan\frac{\left(1+\lambda_{i}\right)\omega_{0}\gamma }{\lambda_{i}},$$ (18)

*where* $\omega_{0}=\sqrt{\frac{{\left(1+\lambda_{i}\right)}^{2}\gamma^{2}\pm \sqrt{{\left(1+\lambda_{i}\right)}^{4}\gamma^{4}+4\lambda_{i}^{2}}}{2}}$ *,* and *λ*_*i*_ *is the characteristic root of the Laplacian matrix* *L* *of the graph* *G* *.*

Proof. For control input $u_{x_{i}}$ , suppose

$$\left\{\begin{array}{c} {\tilde{x}}_{f_{i}}=x_{f_{i}}-\int_{0}^{1}f^{x}\left(s\right)dt-\delta_{i}^{x}\;\\ {\tilde{v}}_{x_{i}}=v_{x_{i}}-f^{x}\left(t\right). \end{array}\right.$$ (19)

According to Eq. 16, there is

$$\left\{\begin{array}{c} {\dot{\tilde{x}}}_{f_{i}}\left(t\right)={\tilde{v}}_{x_{i}}\left(t\right)\;\\ {\dot{\tilde{v}}}_{x_{i}}\left(t\right)=-\gamma {\tilde{v}}_{x_{i}}\left(t-\tau_{ij}\left(t\right)\right)\;\\ -\overset{m}{\underset{j=1}{\sum }}a_{ij}\left[{\tilde{x}}_{f_{i}}\left(t\right)-{\tilde{x}}_{f_{j}}\left(t-\tau_{ij}\left(t\right)\right)\right]\;\\ -\overset{m}{\underset{j=1}{\sum }}a_{ij}\left[\gamma \left({\tilde{v}}_{x_{i}}\left(t-\tau_{ij}\left(t\right)\right)-{\tilde{v}}_{x_{j}}\left(t-\tau_{ij}\left(t\right)\right)\right)\right]. \end{array}\right.$$ (20)

Assuming that all AUVs have the same upper limit of delay *τ*_0_, we have

$$\left[\begin{array}{c} {\dot{\tilde{x}}}_{f}\left(t\right)\\ {\dot{\tilde{v}}}_{x}\left(t\right) \end{array}\right]=\left[\begin{array}{cc} 0 & I_{n}\\ 0 & 0 \end{array}\right]\left[\begin{array}{c} {\tilde{x}}_{f}\left(t\right)\\ {\tilde{v}}_{x}\left(t\right) \end{array}\right]+\left[\begin{array}{cc} 0 & 0\\ -L & -\gamma \left(I_{n}+L\right) \end{array}\right]\left[\begin{array}{c} {\tilde{x}}_{f}\left(t-\tau_{0}\right)\\ {\tilde{v}}_{x}\left(t-\tau_{0}\right) \end{array}\right],$$ (21)

where ${\tilde{x}}_{f}\left(t\right)={\left[{\tilde{x}}_{f_{1}}\left(t\right),\ldots ,{\tilde{x}}_{f_{n}}\left(t\right)\right]}^{T}$ , ${\tilde{v}}_{x}\left(t\right)={\left[{\tilde{v}}_{x_{1}}\left(t\right),\ldots ,{\tilde{v}}_{x_{n}}\left(t\right)\right]}^{T}$ . Suppose $x\left(t\right)={\left[{\tilde{x}}_{f}\left(t\right),{\tilde{v}}_{x}\left(t\right)\right]}^{T}$ , we can get

$$\dot{x}\left(t\right)=\left[\begin{array}{cc} 0 & I_{n}\\ 0 & 0 \end{array}\right]x\left(t\right)+\left[\begin{array}{cc} 0 & 0\\ -L & -\gamma \left(I_{m}+L\right) \end{array}\right]x\left(t-\tau_{0}\right).$$ (22)

A Laplace variation of Eq. 22 is

$$sx\left(s\right)=\left[\begin{array}{cc} 0 & I_{m}\\ 0 & 0 \end{array}\right]x\left(s\right)+\left[\begin{array}{cc} 0 & 0\\ -L & -\gamma \left(I_{m}+L\right) \end{array}\right]e^{-\tau_{0}s}x\left(s\right).$$ (23)

The characteristic equation satisfies

$$\det\left[sI_{2n}-\left[\begin{array}{cc} 0 & I_{n}\\ 0 & 0 \end{array}\right]-e^{-\tau_{0}s}\left[\begin{array}{cc} 0 & 0\\ -L & -\gamma \left(I_{n}+L\right) \end{array}\right]\right]=0,$$ (24)

$$\det\left[\begin{array}{cc} sI_{n} & -I_{n}\\ e^{-\tau_{0}s}L & sI_{n}+e^{-\tau_{0}s}\gamma \left(I_{n}+L\right) \end{array}\right]=0,$$ (25)

$$\det\left(s^{2}I_{m}+e^{-\tau_{0}s}\gamma \left(I_{m}+L\right)s+e^{-\tau_{0}s}L\right)=0.$$ (26)

It follows from Section 2.1 that for an undirected connected graph *G*, the rank of *L* is *rank*(*L*) = *m* − 1; the eigenvalue of *L* is 0 = *λ*_1_ < *λ*_2_ ≤ …, ≤ *λ*_*m*_ = *λ*_max_, then

$$s\overset{m}{\underset{i=2}{\prod }}\left[s^{2}+e^{-\tau_{0}s}\left(\gamma \left(1+\lambda_{i}\right)s+\lambda_{i}\right)\right]=0.$$ (27)

When *i* = 2, … , *m*, Eq. 27 can be organized as

$$1+e^{-\tau_{0}s}\left[\frac{1}{s}\gamma \left(1+\lambda_{i}\right)+\frac{1}{s^{2}}\lambda_{i}\right]=0.$$ (28)

Suppose $G_{i}\left(s\right)=e^{-\tau_{0}s}\left[\frac{1}{s}\gamma \left(1+\lambda_{i}\right)+\frac{1}{s^{2}}\lambda_{i}\right]$ , the number of unstable poles *p* = 0 is consistent with the minimum phase system characteristics. Based on the Nyquist stability criterion (Chou et al., 2020; Wang et al., 2021), the roots of Eq. 28 are in the left half-open plane of the *s*-plane when the Nyquist curve *G*_*i*_(*s*) does not enclose the critical point (−1, *j*0). Then, the system reaches asymptotic consistency. Consequently, when Eq. 18 is satisfied, the roots of Eq. 28 are in the left half-open plane of the s-plane, and the system can reach agreement. At this point, $x_{f_{i}}-x_{f_{j}}\to \delta_{i}^{x}-\delta_{i}^{x}$ , $v_{x_{i}}\to v_{x_{j}}\to f^{x}\left(t\right)$ . Similarly, when control inputs $u_{y_{i}}$ satisfy Eq. 18, $y_{f_{i}}-y_{f_{j}}\to \delta_{i}^{y}-\delta_{i}^{y}$ , $v_{y_{i}}\to v_{y_{j}}\to f^{y}\left(t\right)$ .

### 3.2 Consensus Control Algorithm for Heterogeneous AUV Group Under Communication Delay

Similarly, time delay exists for heterogeneous AUV groups in a natural environment for underwater communication. The following will investigate the consistency control algorithm for the AUV group with both heterogeneity and time delay conditions. Suppose that the state of each AUV in the group is measurable.

In Zheng et al. (2011), the heterogeneous multi-agents are studied. Considering heterogeneous AUV group with *N* linear dynamics, the dynamic model of AUV*i* can be given by Eq. 29.

$${\dot{x}}_{i}\left(t\right)=-Cx_{i}\left(t\right)+Af_{i}\left(x_{i}\left(t\right)\right)+Bf_{i}\left(x_{i}\left(t-\tau_{i}\left(t\right)\right)\right)+u_{i}\left(t\right),$$ (29)

where *i* = 1, 2, … , *N* denotes follower AUV; *x*_*i*_(*t*) ∈ *R* ^*n*^ and *u*_*i*_(*t*) ∈ *R* ^*n*^ are the state vector and control input vector of the system, respectively; *f*_*i*_(*x*_*i*_(*t*)) ∈ *R* ^*n*^ is the unknown continuous non-linear function of the system; and *τ*_*i*_(*t*) > 0 denotes the underwater communication delay. $C=\mathrm{diag}\left\{c_{1},c_{2},\ldots ,c_{n}\right\}\in R^{n\times n}$ , *c*_*i*_ > 0, **A** ∈ *R* ^*n*×*n*^, and **B** ∈ *R* ^*n*×*n*^ are the known system matrices. Each AUV has a different non-linear function *f*_*i*_(*x*_*i*_(*t*)) and time delay *τ*_*i*_(*t*). Different types of AUVs are included by Eq. 29 through selecting different system matrices **A**, **B**, **C**.

Suppose global state vector $x\left(t\right)={\left[x_{1}^{T}\left(t\right),\ldots ,x_{N}^{T}\left(t\right)\right]}^{T}\in R^{nN}$ , Eq. 29 can be written as Eq. (30).

$$\dot{x}\left(t\right)=-\left(I_{N}⊗C\right)x\left(t\right)+\left(I_{N}⊗A\right)f\left(x\left(t\right)\right)+\left(I_{N}⊗B\right)f\left(x\left(t-\tau \left(t\right)\right)\right)+u\left(t\right),$$ (30)

where

$$f\left(x\left(t\right)\right)={\left[f_{1}^{T}\left(x_{1}\left(t\right)\right),\ldots ,f_{N}^{T}\left(x_{N}\left(t\right)\right)\right]}^{T}\in R^{nN},$$ (31)

$$f\left(x\left(t-\tau \left(t\right)\right)\right)={\left[f_{1}^{T}\left(x_{1}\left(t-\tau_{1}\left(t\right)\right)\right),\ldots ,f_{N}^{T}\left(x_{N}\left(t-\tau_{N}\left(t\right)\right)\right)\right]}^{T}\in R^{nN},$$ (32)

$$u\left(t\right)={\left[u_{1}^{T}\left(t\right),\ldots ,u_{N}^{T}\left(t\right)\right]}^{T}\in R^{nN}.$$ (33)

The dynamic system of the leader (AUV0) is as follows:

$${\dot{x}}_{0}\left(t\right)=-Cx_{0}\left(t\right)+Af_{0}\left(x_{0}\left(t\right)\right)+Bf_{0}\left(x_{0}\left(t-\tau_{0}\left(t\right)\right)\right),$$ (34)

where *x*_0_(*t*), *f*_0_(*x*_0_(*t*)), and *τ*_0_(*t*) are the leader’s state vector, non-linear function vector, and time-varying system delay, respectively. Similarly, the leader node 0 can be used as an external reference system or command controller to generate the required dynamic trajectory. Suppose that the leader node could not be affected by *n* follower nodes.

Suppose that only information on neighboring nodes is available, local consistency error for AUV*i* is shown as follows:

$$e_{i}\left(t\right)=\overset{N}{\underset{j=1}{\sum }}a_{ij}\left(x_{j}\left(t\right)-x_{i}\left(t\right)\right)+g_{i}\left(x_{0}\left(t\right)-x_{i}\left(t\right)\right).$$ (35)

If AUV*i* can get the state information of AUV*j*, the connection weight *a*_*ij*_ > 0 and the traction gain *g*_*i*_ > 0. Otherwise, *a*_*ij*_ = 0 and *g*_*i*_ = 0. Combined with the consistency control strategy, the following control law is designed to achieve the control objective of the AUV group.

$$u_{i}\left(t\right)=cKe_{i}\left(t\right)-A\varphi_{i}\left(x_{i}\left(t\right)\right)-B\left(t-\tau_{im}\right)\varphi_{i}\left(x\left(t-\tau_{im}\right)\right),$$ (36)

where *c* > 0, *K* ∈ *R* ^*n*×*n*^ are the pending control gain and feedback gain matrices, respectively, and *τ*_*im*_ denotes the upper bound of the unknown time delay *τ*_*i*_(*t*). Consistent stability proofs refer to Section 3.1.

## 4 Simulation and Analysis

**FIGURE 3:**
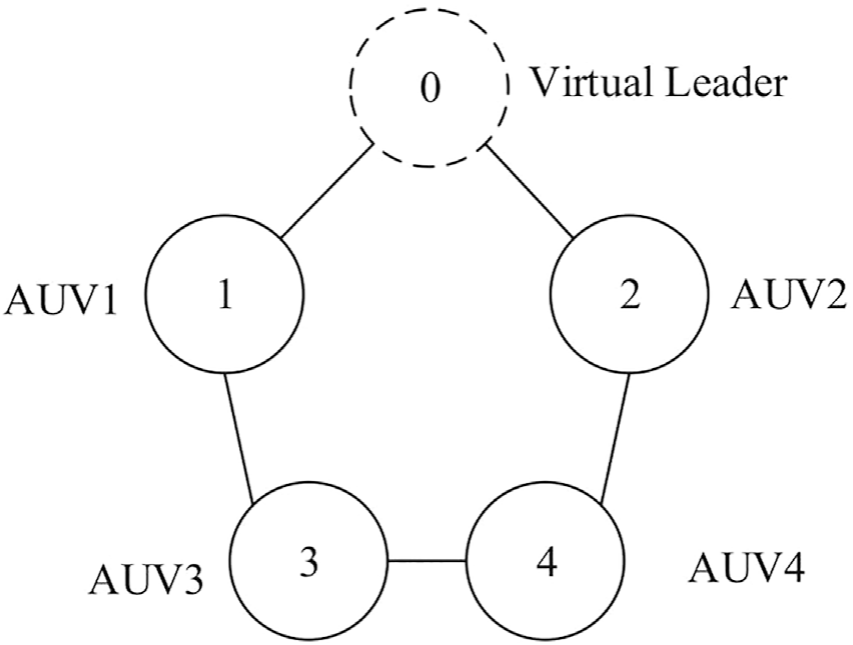
Communication topology diagram.

In this section, one leader and four followers are set to form an AUV group. The initial position of the leader is randomly distributed between (−4, 4), the initial position of each follower is randomly distributed in the interval (−8, 8), the initial combined speed is 5 m/s, and the initial values of other state variables are set to 0. The communication topology diagram is defined as an undirected connectivity diagram, which is shown in Figure 3.

The system weighted adjacency matrix is shown as follows:

$$A=\left[\begin{array}{ccccc} 0 & 1 & 1 & 0 & 0\\ 1 & 0 & 0 & 1 & 1\\ 1 & 0 & 0 & 0 & 1\\ 0 & 1 & 0 & 0 & 0\\ 0 & 1 & 1 & 0 & 0 \end{array}\right]B=\left[\begin{array}{ccccc} 2 & 0 & 0 & 0 & 0\\ 0 & 3 & 0 & 0 & 0\\ 0 & 0 & 2 & 0 & 0\\ 0 & 0 & 0 & 1 & 0\\ 0 & 0 & 0 & 0 & 2 \end{array}\right].$$

**FIGURE 4:**
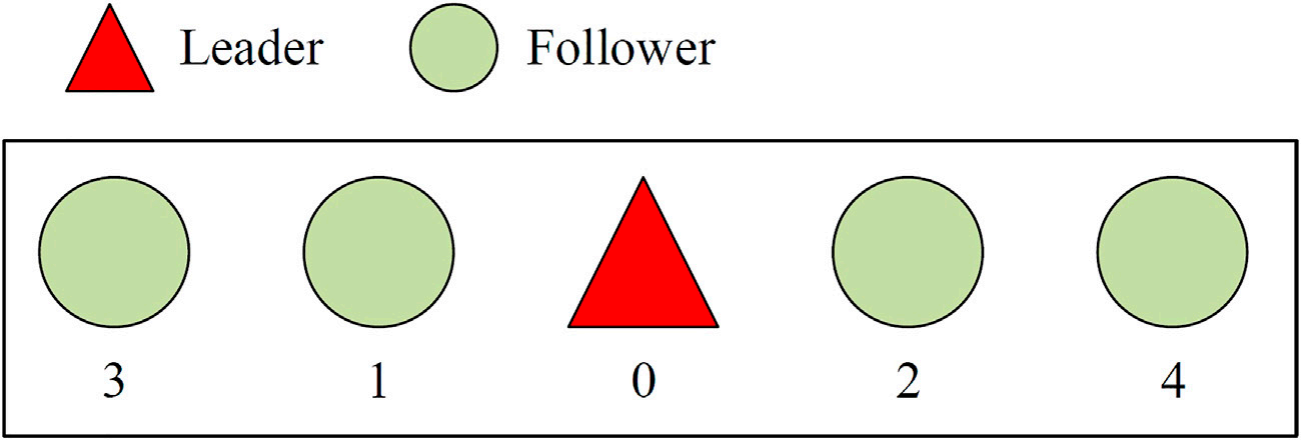
Parallel formation diagram.

As shown in Figure 4, a parallel formation is designed with the leader as the center and four followers evenly distributed around it. The triangle in the figure represents the leader, and the circle represents the follower. The control gain factor can be set as *γ*_*x*_ = *γ*_*y*_ = 2, *γ*_*z*_ = 1.

### 4.1 Simulation of Homogeneous AUV Group Under Communication Delay

In this section, a simulation experiment is conducted for the consistency control algorithm with communication delay proposed in Section 3.1. The difference is that the communication time delay is set to *τ* = 0.5.

**FIGURE 5:**
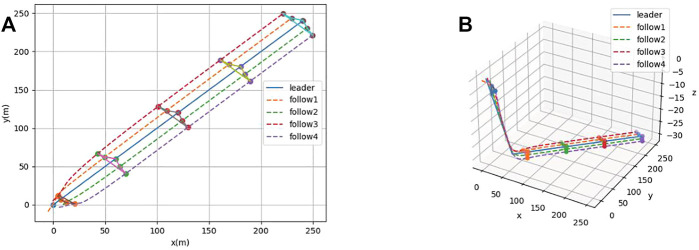
**(A)** Formation process under communication delay (2-D). **(B)** Formation process under communication delay (3-D).

**FIGURE 6:**
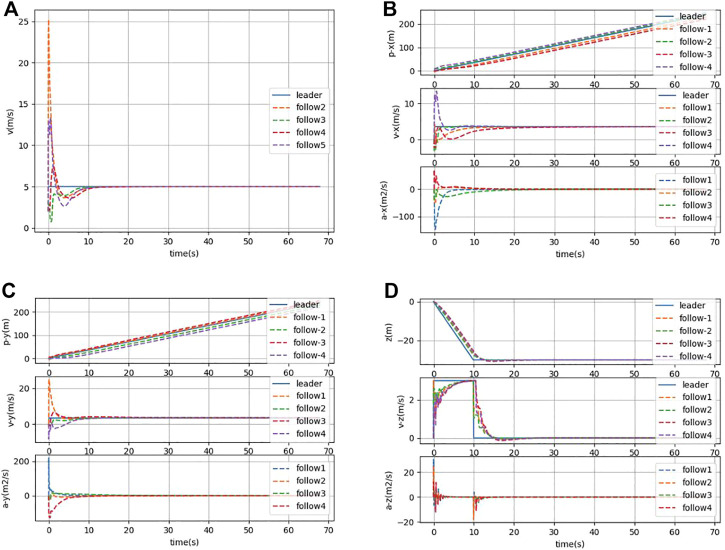
**(A)** Combined velocity in the *x*, *y*, and *z* directions under communication delay. **(B)** Position, velocity, and acceleration states in the *x*-direction under communication delay. **(C)** Position, velocity, and acceleration states in the *y*-direction under communication delay. **(D)** Position, velocity, and acceleration states in the *z*-direction under communication delay.

In Figure 5, it can be seen that the AUV group has a lag in the state of the follower in the condition of the presence of communication delay. The followers cannot keep a horizontal line with the leader, which means that the parallel formation cannot be maintained. However, the AUV group can still maintain a steady-state moving forward. In Figure 6, it can be seen that under the condition of communication time delay, the velocity of each follower AUV in *x*, *y*, and *z* directions is jittered and then converges rapidly. Simultaneously, the acceleration of each follower finally converges to zero.

### 4.2 Simulation of Heterogeneous AUV Group Under Communication Delay

**FIGURE 7:**
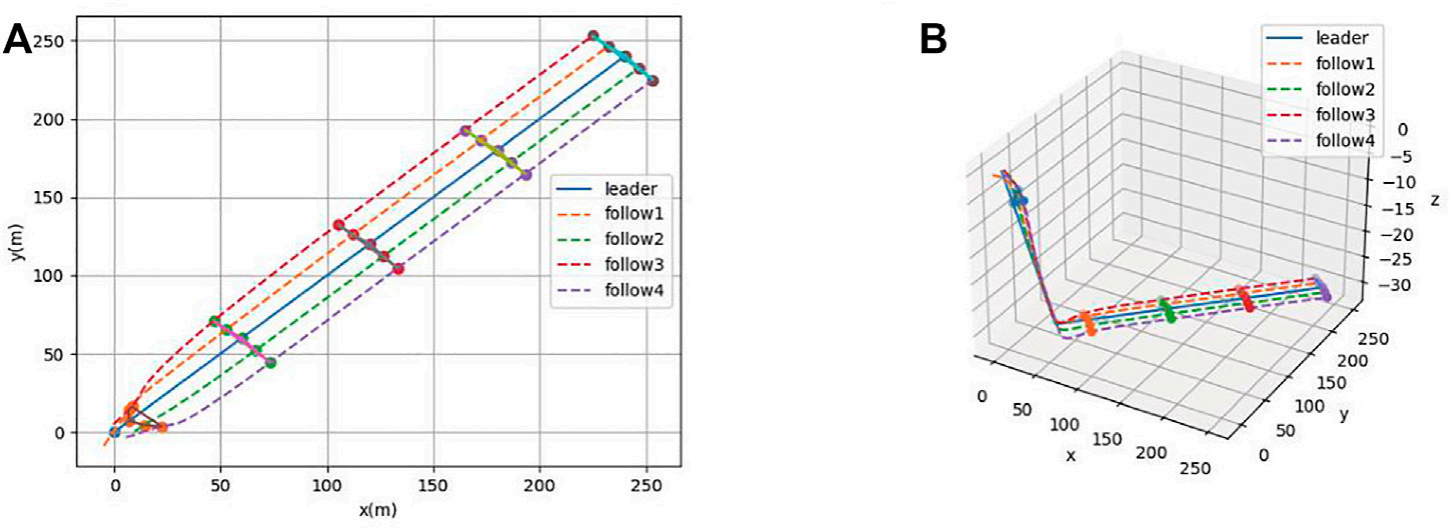
**(A)** Formation process the heterogeneous AUV group under communication delay (2-D). **(B)** Formation process the heterogeneous AUV group under communication delay (3-D).

**FIGURE 8:**
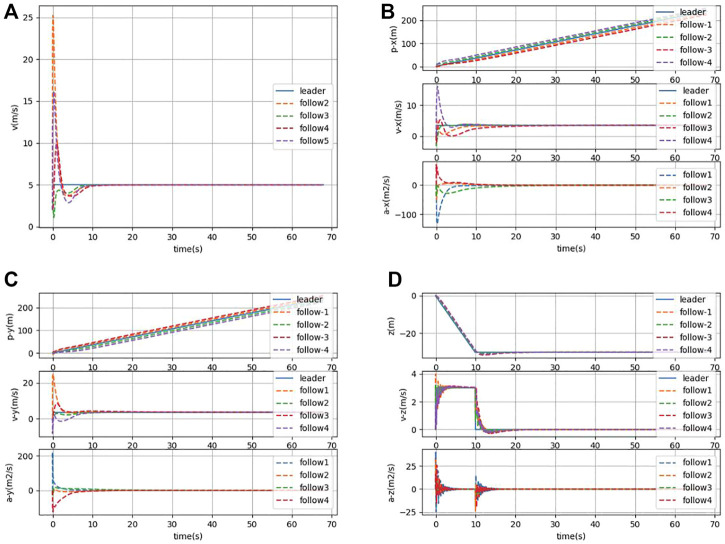
**(A)** Combined velocity of the heterogeneous AUV group in the *x*, *y*, and *z* directions under communication delay. **(B)** Position, velocity, and acceleration states of the heterogeneous AUV group in the *x*-direction under communication delay. **(C)** Position, velocity, and acceleration states of the heterogeneous AUV group in the *y*-direction under communication delay. **(D)** Position, velocity, and acceleration states of the heterogeneous AUV group in the *z*-direction under communication delay.

A simulation experiment is conducted for the consistency control algorithm in Section 3.2. Different types of AUVs are included in Eq. 29 by selecting different system matrices **C**. Figure 7 and Figure 8 show the formation process and the state of formation keeping navigation of the heterogeneous AUV group through the horizontal plane and three-dimensional views, respectively. Compared with Section 3.1, the specified formation is also formed at around *t* = 20*s*, which indicates that the formation process remains constant when the control gain remains the same. Moreover, the latency of the simulation is much smaller than that of Section 4.1. Therefore, the effectiveness of the control algorithm of the heterogeneous AUV group under communication delay is proved.

The distributed controller designed in this study can be applied to the formation control of heterogeneous AUV groups. Under the condition of time delay existence, the control protocol does not need to know the specific form of complex non-linear functions in each AUV motion model and also does not need to know the unknown time-varying time delay existing in the system; it needs to know only the neighbor information of local AUVs within the heterogeneous AUV network. In addition, the corresponding control gain can be derived off-line for a determined heterogeneous AUV group. Therefore, the proposed control scheme can be easily implemented in terms of design and implementation. The simulation results show that under the conditions of unknown time-varying time delay, variable network topology, and intermittent communication, all the followers can still converge to the leader’s trajectory quickly and exhibit their heterogeneous characteristics as well as the leader.

## 5 Conclusion

In this study, a formation control method of the homogeneous and the heterogeneous AUV group combining the consensus theory and leader–follower method under communication delay is proposed. A hybrid communication topology that can be applied to the formation control of a large AUV group is established. Moreover, a virtual leader is used to overcome the over-reliance on the leader. The simulation results show that the consistency control algorithm designed in this study can make all follower AUVs follow the leader for stable motion under both communication delay and no communication delay. In the future, a compensator will be designed to overcome interference in a complex ocean environment.

## Data Availability

The original contributions presented in the study are included in the article/Supplementary Material, further inquiries can be directed to the corresponding author.
